# A Contribution to the Pathogenesis of Flint’s Murmur

**Published:** 1913-11

**Authors:** 


A CONTRIBUTION TO THE PATHOGENESIS OF FLINT'S MURMUR. M. Carrieu and J. Anglada, Montpelier, *Arch. des Mal. du Coeur, des Vaisseaux, et du Sang.,* 1913, VI, 253. Carrieu and Anglada report a case of aortic regurgitation with a distinct Flint murmur, with autopsy. The Flint murmur was due to a spur which projected into the left auricle from the wall of the aorta, encroaching upon the mitral orifice. The mitral valve leaflets were normal.

